# 

**Published:** 1861-10

**Authors:** 


ORIGINAL ESSAYS AND COMMUNICATIONS.

Labor. By W. H. Atkinson, D. D. S. .......... 533

PROCEEDINGS OF SOCIETIES.

American Dental Convention, .......... 539

SELECTIONS.

A New Anæsthetic Kerosolene, .......... 570
New Anæsthetics.—Theory of their Action, .......... 572

EDITORIAL.

Temporal Economy, .......... 575
Bleaching, .......... 576
Postponement, .......... 578
Personal, .......... 578
Tricks of the Types, .......... 578
Diseased Antrum, .......... 579
A Model Patient, .......... 580
Hemorrhage, .......... 580
Wood for Finishing Fillings, .......... 580

